# Evaluating nursery pig responses to in-feed sub-therapeutic antibiotics

**DOI:** 10.1371/journal.pone.0216070

**Published:** 2019-04-26

**Authors:** Emma T. Helm, Shelby Curry, Julian M. Trachsel, Martine Schroyen, Nicholas K. Gabler

**Affiliations:** 1 Department of Animal Science, Iowa State University, Ames, Iowa, United States of America; 2 Oak Ridge Institute for Science and Education, Oak Ridge, Tennessee, United States of America; 3 Food Safety and Enteric Pathogens Research Unit, National Animal Disease Center, Agriculture Research Service, United States Department of Agriculture, Ames, Iowa, United States of America; 4 Precision Livestock and Nutrition Unit, TERRA Teaching and Research centre, Gembloux Agro-Bio Tech, University of Liège, Gembloux, Belgium; University of Illinois, UNITED STATES

## Abstract

Antibiotics have been used for over 60 years by the swine industry to improve growth performance and feed efficiency. With rising concerns over antimicrobial resistance and government restrictions such as the Veterinary Feed Directive on usage of in-feed antibiotics, alternatives to feeding antibiotic growth promoters (**AGPs**) to nursery pigs are needed. However, the mechanism of action by which AGPs work is poorly understood. Thus, the objective of this study was to investigate the mechanisms of action by which AGPs increase nursery pig performance. Over two replicates, 24 weaned pigs (6.75 ± 0.75 kg body weight) were randomly allotted to either control (**CON**, n = 12) or sub-therapeutic antibiotic (**sCTC**, n = 12) treatments and housed individually. A 2-phase corn-soybean-based nursery diet was fed, with the sCTC diets containing 40 ppm feed-grade chlortetracycline. Individual pig average daily gain (**ADG**), average daily feed intake (**ADFI**), and gain to feed ratio (**G:F**) were calculated weekly for 5 weeks. Thereafter, all pigs were euthanized and necropsied for tissue collection. The overall performance data indicated that sCTC pigs had increased ADG (0.43 vs. 0.32 kg/d, *P* = 0.001) and ADFI (0.51 vs. 0.37 kg/d, *P* = 0.002) compared with CON pigs; however, G:F was not different as a result of dietary treatment (0.85 vs. 0.88, *P* = 0.617). Intestinal barrier permeability, ileal active nutrient transport, and cecal short chain fatty acid concentrations did not differ (P > 0.10) due to dietary treatment, however changes in several ileum mRNA transcripts suggest that inflammation may be reduced in sCTC pigs. Further, the changes observed in the proteomes of the ileum, colon, skeletal muscle, and liver suggest that the sub-therapeutic mode of action of AGPs may include post-absorptive changes and warrants further investigation.

## Introduction

Sub-therapeutic antibiotic growth promotants (**AGPs**) have been used heavily by the swine industry since their growth promoting qualities were first discovered in the 1940s [1]. Antibiotic growth promotants have been shown to consistently improve body weight gain and feed efficiency in growing pigs, specifically during the nursery phase [2]. However, rising concerns over antimicrobial resistance to antibiotics has resulted in the United States animal agriculture sector banning the use of sub-therapeutic growth promoting antibiotics via the recent implementation of the 2017 Veterinary Feed Directive (**VFD**) [3]. The VFD prohibits the usage of sub-therapeutic AGPs, which can be defined an antimicrobial fed at concentrations lower than the lowest concentration that will inhibit the growth of the target microorganism of that antimicrobial. One of the most commonly used AGPs has been chlortetracycline (**CTC**) [4]. The potential of tetracycline antibiotics to promote growth was first shown in the 1940s when healthy animals that consumed dried mycelia of *Streptomyces aureofaciens* containing chlortetracycline residues were observed to have improved growth [5]. The tetracycline class of antibiotics exert their effect on Gram-positive, and to a lesser extent, Gram-negative bacteria by binding to the bacterial 30S and 50S ribosome and halting protein synthesis [6, 7]. In hosts, tetracyclines have also been shown to act as reactive oxygen species scavengers or anti-inflammatory agents, to inhibit matrix metalloproteinases, and to possess anti-apoptotic properties [6]. Tetracycline antibiotics have been used both therapeutically and sub-therapeutically in animal agriculture. In-feed therapeutic levels of CTC have historically been 400 ppm or greater [8]. For use as an in-feed sub-therapeutic AGP, CTC has been added at low concentrations (2.5 ppm to 125 ppm), depending upon both the animal species and the drug type, to enhance animal performance as opposed to treating, controlling, and preventing disease [9, 10].

The loss of a major production tool has created a need by animal production industries to find alternatives that mimic the beneficial performance aspect of AGPs while minimizing the potential for antibiotic resistance. Despite their heavy usage in the livestock industry, the mechanism by which AGPs increase growth performance is still largely unknown. The hypothesis that has received the most attention proposes that AGPs act indirectly on the pig via modulation of the intestinal microbiota. This hypothesis suggests that AGPs reduce production of growth-depressing metabolites by microbes, suppress pathogenic microbial growth, and reduce competition for nutrients to allow for heightened nutrient uptake [11]. The hypothesis is best supported by the observation that antibiotics don’t improve the growth of germfree chicks [12]; however, a clear and direct link between AGPs and modulation of the intestinal microbiota has not yet been demonstrated [9].

Another hypothesis postulates that AGPs act via a more direct effect on the host. This hypothesis primarily revolves around immunomodulation of the gastrointestinal tract (**GIT**), in which AGPs exert anti-inflammatory properties on the intestine, resulting in more energy being available for lean tissue growth rather than maintaining an immune response [13]. Although studies investigating the immunomodulatory hypothesis are limited, Costa et al. [13] demonstrated that sub-therapeutic levels of CTC altered the immune response to *Citrobacter rodentium* infection in mice, consistent with the immunomodulatory hypothesis.

In order to develop better in-feed technologies to replace AGPs such as CTC, one must first understand how they act on the host to improve growth. Therefore, the aim of this study was to examine the mechanism(s) by which in-feed sub-therapeutic CTC enhances the growth performance of nursery pigs. A whole body, shotgun proteomic and PCR array approach was utilized to identify potential mechanisms of action by which CTC improves growth performance of nursery pigs.

## Materials and methods

All animal procedures in this study were approved by the Iowa State University Institutional Animal Care and Use Committee (protocol number 4-16-8251-S) and adhered to the ethical and humane use of animals for research.

### Animals, housing, and experimental design

This experiment was performed in two experimental replicates in the summer and autumn of 2016. A total of 24 weaned female pigs (Genetiporc 6.0 × Genetiporc F25; PIC, Inc., Hendersonville, TN), 19–21 days of age (6.75 ± 0.75 kg body weight), were randomly selected for this experiment. Pigs were vaccinated for PCV2 prior to weaning. After selection, pigs were randomly allotted to individual pens and assigned to one of two dietary treatments: 1) negative control group (**CON**, n *=* 12 pigs/trt), and 2) sub-therapeutic antibiotic treatment (**sCTC**, n *=* 12 pigs/trt). The sCTC treatment consisted of the basal CON diet mixed with feed-grade CTC (Zoetis, Parsippany, NJ) to achieve a final sub-therapeutic level of 40 ppm in feed. This sub-therapeutic concentration was chosen as it was the manufacturer’s recommended inclusion rate for growth promotion. Both treatment diets (**Table 1**) were fed as a two phase nursery mash diet formulated to meet all requirements for this size pig [14]. The phase 1 diet was fed from days 0–14 of the experiment, and the phase 2 diet was fed from days 14–35 of the experiment. Pigs were allowed free access to water and were *ad libitum* fed for the duration of the experiment.

**Table 1:** Diet composition, as fed.

	CON		sCTC	
Ingredient, %	Phase 1	Phase 2	Phase 1	Phase 2
Corn	52.38	60.07	52.38	60.07
Soybean meal, 46.5% CP	22.00	27.88	22.00	27.88
Casein	5.37	2.50	5.37	2.50
Lactose	10.00	2.50	10.00	2.50
Fish meal	5.00	2.50	5.00	2.50
Soybean oil	1.77	1.57	1.77	1.57
L-lysine HCl	0.29	0.19	0.29	0.19
DL-methionine	0.13	0.06	0.13	0.06
L-threonine	0.11	0.04	0.11	0.04
L-valine	0.20	0.00	0.20	0.00
Monocalcium phosphate, 21%	1.03	0.86	1.03	0.86
Limestone	0.82	0.93	0.82	0.93
Salt	0.50	0.50	0.50	0.50
Vitamin premix^1^	0.25	0.25	0.25	0.25
Mineral premix^2^	0.15	0.15	0.15	0.15
Chlortetracycline, active ingredient	—	—	0.04	0.04
*Calculated composition*				
CP, %	23.07	21.98	23.07	21.98
ME, kcal/kg	3,410	3,388	3,410	3,388
NE, kcal/kg	2,519	2,453	2,519	2,453
Lys, SID^3^ %	1.50	1.29	1.50	1.29

^1^Provided per kilogram of diet: 6,125 IU vitamin A, 700 IU vitamin D_3_, 50 IU vitamin E, 30 mg vitamin K, 0.05 mg vitamin B_12_, 11 mg riboflavin, 56 mg niacin, and 27 mg pantothenic acid.

^2^Provided per kilogram of diet: 22 mg Cu (as CuSO_4_), 220 mg Fe (as FeSO_4_), 0.4 mg I (as Ca(IO_3_)_2_), 52 mg Mn (as MnSO_4_), 220 mg Zn (as ZnSO_4_), and 0.4 mg Se (as Na_2_SeO_3_).

^3^SID = standardized ileal digestibility

On days 0, 7, 14, 21, 28, and 35 post-weaning, individual pig body weight and feed disappearance were recorded to calculate average daily gain (**ADG**), average daily feed intake (**ADFI**) and gain:feed (**G:F**) ratio. At day 35, 6 pigs per treatment were euthanized via captive bolt followed by exsanguination and tissues were collected for analysis. Immediately following euthanasia, sections from the ileum and colon were flushed of luminal contents with Krebs-Henseleit buffer (**KB**; 25 mM NaHCO_3_, 120 mM NaCl, 1 mM MgSO_4_, 6.3 mM KCl, 2 mM CaCl_2_, and 0.32 mM NaH_2_PO_4_, pH 7.4) and placed into aerated bottles containing KB for transportation and mounting into modified Ussing chambers (Physiological Instruments and VCC MC8; World Precisions Instruments, New Haven, CT). Additionally, sections from the ileum, colon, *Longissimus dorsi* muscle (**LM**), and liver as well as contents from the cecum were frozen in liquid nitrogen and stored at -80°C until analysis.

#### Intestinal integrity and function

Fresh ileum and colon segments were mounted into modified Ussing chambers (Physiological Instruments, San Diego, CA and World Precision Instruments, Sarasota, FL) to determine intestinal barrier integrity and ileal active nutrient transport. Tissues were pinned and placed vertically into chambers, connected to dual channel voltage and current electrodes submerged in 3% noble agar, and filled with 3 M KCl to provide electrical conductance. The serosal and mucosal sides were bathed in 4 mL KB. Tissue segments were provided with a constant O_2_-CO_2_ mixture. Each segment was clamped at a voltage of 0 mV after correction for solution resistance. A pulse current was applied and transepithelial electrical resistance (**TER**) measurements were calculated based on measured voltage and the change in short circuit current when the current pulse was applied. The TER was averaged over 10 a minute collection period after 10 minutes of stabilization. After 30 and 45 minutes, ileal tissues were independently challenged with the mucosal addition of 10 mM D-glucose and 10 mM L-glutamine, respectively [15]. Equimolar (10 mM) mannitol was concurrently added to the serosal chamber with each challenge. The maximal current was recorded after each challenge, subtracted from the current immediately prior each challenge, and the change in short circuit current (μA) was calculated.

### Cecal short chain fatty acid concentrations

To evaluate short chain fatty acid (SCFA) concentrations, 1 g of frozen cecal contents were suspended in 2 mLphosphate buffered saline, vortexed for one minute, and debris was pelleted by centrifugation at 5000 × *g* for 10 minutes. The resulting supernatant (1 mL) was added to heptanoic acid internal standards. Butylated fatty acid esters were generated as described [16], analyzed using an Agilent 7890 GC (Agilent, Santa Clara, CA), and data were reported as a concentration of short chain fatty acid (mM) per gram of cecal content.

### Ileum mRNA abundance

Total RNA was extracted from frozen ileum samples utilizing the Trizol protocol (Invitrogen, Grand Island, NY). Quantity and purity of extracted RNA was determined using a NanoDrop 1000 (NanoDrop Technologies, Rockland, DE). All samples had a 260/280 ratio of at least 1.8. One microgram of extracted RNA was transcribed using a commercially available kit (Quantitect reverse transcription kit; Qiagen Inc., Valencia, CA) and resulting cDNA was quantified using a Nanodrop 1000 and utilized for quantitative real-time PCR (**qPCR**) using a BioMark HD system (Fluidigm Corporation, San Francisco, CA). Complementary DNA was amplified using the TaqMan PreAmp Master Mix (Life Technologies, Carlsbad, CA) and loaded onto Fluidigm’s Dynamic Array Integrated Fluidic Circuits following Fluidigm’s EvaGreen DNA binding dye protocol. Gene symbols and primer sequences are listed in S1 Table. The endogenous reference genes RPL32, ACTB, and TOP2B were included in the array for standardization. The mRNA abundance values for each sample were normalized to reference genes and CON pigs according to the 2^-ΔΔCt^ method.

### Proteomics (liquid chromatography-mass spectrometry)

To evaluate tissue protein abundance changes that may occur due to sCTC supplementation, the proteomes of the ileum, colon, LM, and liver were evaluated at 35 days post-weaning. Proteins were analyzed at the Iowa State University Protein Facility (Ames, IA) using a Q-Exactive Hybrid Quadrupole-Orbitrap Mass Spectrometer (ThermoFisher Scientific, Waltham, MA). Proteins were extracted from frozen tissue and digested in trypsin/Lys-C. After digestion, 250 fmol of peptide retention time calibration (**PRTC**) standard (ThermoFisher Scientific, Waltham, MA) was spiked into each sample as an internal control. Peptides were separated by liquid chromatography and analyzed via MS/MS by fragmentation of peptides. The intact and fragmentation pattern was compared to a theoretical fragmentation pattern using Mascot software (Matrix Science, Boston, MA) to find peptides that were used to identify the proteins against the *Sus scrofa* Swiss-Prot database. Sequest HT software was utilized to run samples against the PRTC database. Label-free quantification, using Minora Feature Detector, was used to detect and quantify isotopic clusters. The PRTC areas were used to normalize data between samples. Identified proteins were sorted by Mascot score, and any protein with a Mascot score <100 was removed from analysis. Abundances were tabulated after calculating the log_2_ change in sCTC pig proteins compared with CON proteins.

### Statistical analysis

Statistical analysis was performed in SAS 9.4 (SAS Institute Inc., Cary, NC). Pig performance, intestinal TER, active transport of glucose and glutamine, cecum VFA concentrations production, and Fluidigm data were analyzed using the MIXED procedure to examine dietary treatment effects. The model included a random effect of replicate. Growth performance parameters (ADG, ADFI, and G:F) were analyzed as time repeated measures over the duration of the study.

Protein abundances were quantified via label-free techniques. Statistical significance between treatments was calculated utilizing a Student’s 2-tailed *t*-test. All data are reported as LSmeans and a pooled standard error of the mean (SEM). Significance was determined when *P* < 0.05 and a tendency when 0.05 ≤ *P* < 0.10.

## Results

### Growth performance

Supplementation of sCTC at a rate of 40 ppm increased (*P* = 0.001, **Table 2**) ADG by 26% compared with pigs fed the CON diet. As such, sCTC pigs were 4 kg heavier (*P* = 0.002) than CON pigs at 35 days post weaning. This increase in ADG appeared to be primarily feed intake driven, as sCTC pigs had 28% greater (*P* = 0.002) ADFI compared with CON pigs, and G:F did not differ (*P* > 0.10) between treatments. These changes associated with sCTC were observed in both dietary phases.

**Table 2:** Growth performance parameters of pigs in either control (CON) or sub-therapeutic chlortetracycline (sCTC; 40 ppm) treatments.

Item	CON^1^	sCTC^1^	SEM	*P*-value
Start body weight, kg	6.64	6.85	0.362	0.444
End body weight, kg	17.55	21.53	0.807	0.002
Day 0–14 ADG^2^, kg/d	0.05	0.16	0.028	0.013
Day 0–14 ADFI^3^, kg/d	0.19	0.25	0.054	0.051
Day 0–14 Gain:Feed	-0.24	0.15	0.449	0.115
Day 15–35 ADG^2^, kg/d	0.50	0.60	0.079	0.001
Day 15–35 ADFI^3^, kg/d	0.67	0.82	0.074	0.002
Day 15–35 Gain:Feed	0.65	0.50	0.228	0.252
Overall ADG^2^, kg/d	0.32	0.43	0.021	0.001
Overall ADFI^3^, kg/d	0.37	0.51	0.044	0.002
Overall Gain:Feed	0.88	0.85	0.096	0.617

^1^Mean values are pig estimates (n = 12 pigs/treatment). Days 0–14 = Diet phase 1, days 15–35 = diet phase 2, and overall equates to days 0 to 35.

^2^ADG = Average Daily Gain

^3^ADFI = Average Daily Feed Intake

### Intestinal permeability, nutrient uptake, microbial fermentation, and gene abundance

Ileum and colon TERs and ileum active nutrient transport did not differ (*P* > 0.10) between treatments (**Table 3**). Additionally, cecal SCFA concentrations did not differ (*P* > 0.10) between treatments for either individual or total SCFA (**Table 4**).

**Table 3:** *Ex vivo* markers of intestinal permeability and nutrient uptake of pigs in either control (CON) or sub-therapeutic chlortetracycline (sCTC; 40 ppm) treatments.

Parameter	CON^1^	sCTC^1^	SEM	*P*-value
Ileum				
TER^2^^,^ ^3^	1.00	0.98	0.041	0.742
Glucose, ΔμA^4^	6.41	6.24	2.251	0.958
Glutamine, ΔμA^4^	1.67	0.77	0.749	0.407
Colon				
TER^2^^,^ ^3^	1.00	1.51	0.578	0.535

^1^Mean values are pig estimates (n = 6 pigs/trt)

^2^TER = transepithelial resistance

^3^Arbitary units

^4^Active absorption calculated by subtracting μA before substrate (glucose or glutamine) from μA after substrate addition.

**Table 4:** Cecal short chain fatty acid concentrations (mM/g cecal content) of pigs in either control (CON) or sub-therapeutic chlortetracycline (sCTC; 40 ppm) treatments.

Parameter	CON^1^	sCTC^1^	SEM	*P*-value
Acetate	76.0	80.3	6.892	0.547
Butyrate	15.4	13.1	2.139	0.465
Caproate	0.07	0.12	0.047	0.469
Formate	0.61	1.11	0.330	0.297
Isobutyrate	0.56	0.36	0.130	0.293
Isovalerate	0.58	0.43	0.129	0.444
Lactate	0.03	0.75	0.367	0.196
Oxalate	0.33	0.27	0.103	0.665
Phenylacetate	0.14	0.08	0.051	0.477
Propionate	39.2	34.2	2.336	0.158
Succinate	0.43	0.46	0.086	0.810
Valerate	2.85	2.29	0.820	0.641
Branch chain fatty acids	1.14	0.79	0.253	0.348
Total SCFA^2^	136.2	133.5	8.015	0.812

^1^Mean values are pig estimates (n = 6 pigs/trt)

^2^SCFA = short chain fatty acid

The abundance of the antimicrobial peptide β-defensin 2 (**BD2**) tended to be reduced (43%, *P* = 0.060) in sCTC pigs compared with CON pigs. Caspase-6 (**CASP6**), a proteolytic protein, was of 45% greater (*P* = 0.030) abundance in sCTC pigs compared with CON pigs (**Table 5**). The abundance of cytokine chemokine ligand 2 (**CCL2**) tended to be reduced (*P* = 0.074) in sCTC pigs compared with CON pigs. Additionally, the abundance of anti-inflammatory cytokine interleukin (**IL**)-10 tended to be reduced (*P* = 0.064) in sCTC pigs compared with CON pigs. However, the abundance of all other genes measured in the ileum did not differ (*P* > 0.10) between treatments.

**Table 5:** Gene abundance in the ileum of pigs in either control (CON) or sub-therapeutic chlortetracycline (sCTC; 40 ppm) treatments.

Parameter^2^	CON^1^	sCTC^1^	SEM	*P*-value
BD2	0.87	0.50	0.505	0.060
CASP3	2.42	3.14	1.766	0.350
CASP6	1.11	2.03	0.270	0.030
CCL2	1.06	0.73	0.123	0.074
CLDN2	1.15	1.02	1.707	0.823
CLDN3	1.93	2.52	0.900	0.639
CLDN4	2.11	2.14	1.551	0.961
DEFB1	0.62	0.86	0.175	0.319
FABP1	3.22	4.54	1.524	0.553
IAP	5.60	4.89	3.328	0.639
IL10	0.92	0.56	0.138	0.064
IL18	0.50	0.57	0.499	0.760
IL8	1.25	0.94	0.285	0.453
MUC2	1.51	1.69	0.857	0.645
NFKB1	1.12	1.02	0.143	0.633
OCLN	2.28	2.42	1.387	0.812
RELA	1.78	1.96	0.226	0.213
SLC5A1	4.06	4.41	2.094	0.719
SLC5A8	2.62	2.85	1.561	0.907
SLCA2	1.54	2.12	1.377	0.314
TFF2	1.18	1.04	0.434	0.491
TGFB1	0.98	0.13	0.134	0.119
TLR2	0.96	0.99	0.612	0.920
TLR3	1.22	1.15	0.361	0.895
TLR4	0.60	0.55	0.132	0.780
TNFA	1.05	0.84	0.879	0.497

^1^Mean values are pig estimates (n = 6–8 pigs/trt)

^2^Gene abundances expressed as fold changes from CON average (2^-ΔΔCt^)

### Protein profile

**Fig 1:**
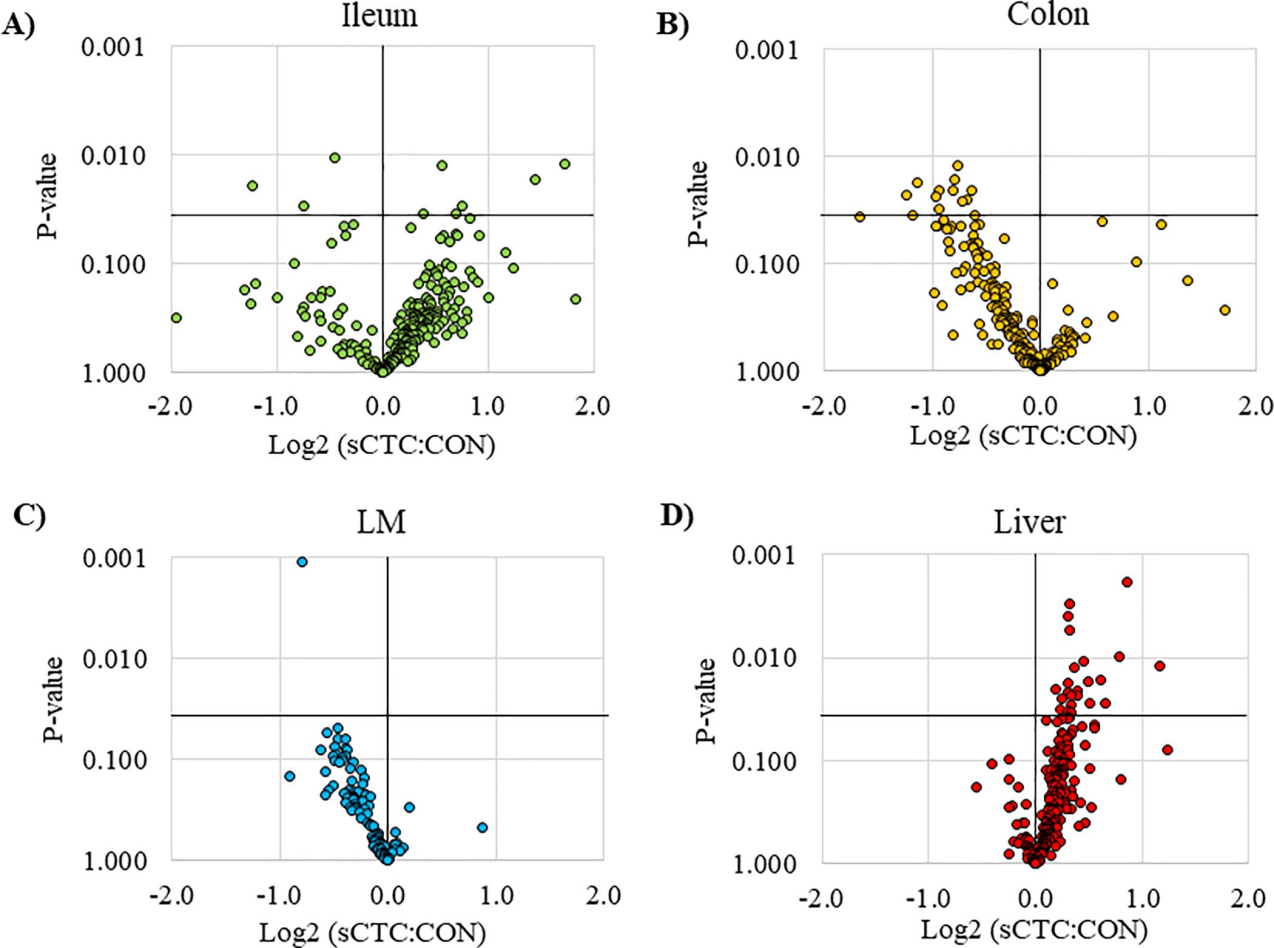
Pairwise proteome comparisons in the **A**) ileum, **B**) colon, **C**) longissimus muscle (LM), and **D**) liver of pigs in control (CON) and sub-therapeutic chlortetracycline (sCTC; 40 ppm) treatments. Proteins identified are demonstrated in relation to average ion intensity differences and *P-*value (statistical significance).

**Fig 2:**
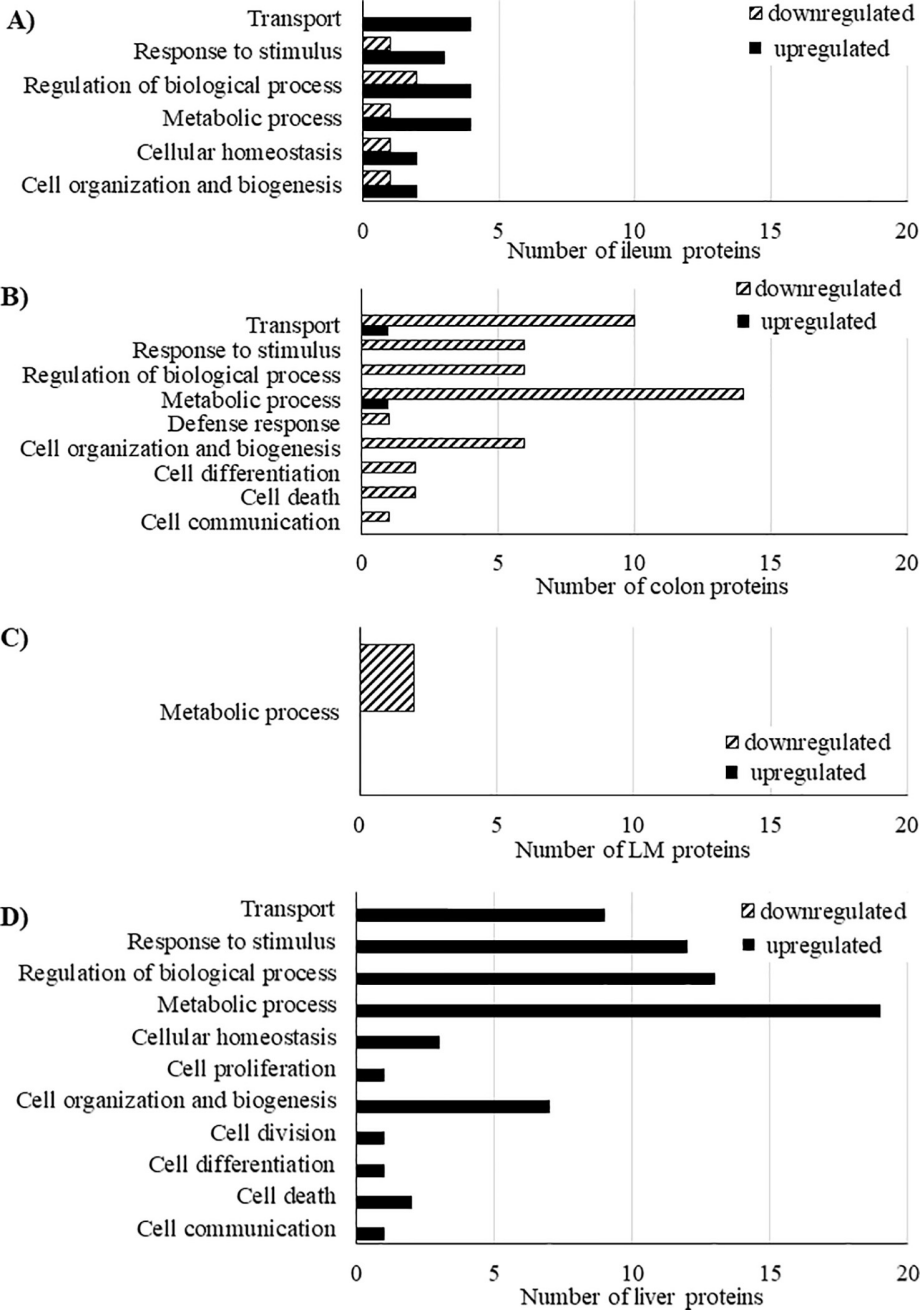
Biological processes of differentially abundant proteins in in the **A**) ileum, **B**) colon, **C**) longissimus muscle, and **D**) liver of pigs in sub-therapeutic chlortetracycline (sCTC; 40 ppm) compared with control (CON) treatments.

The protein profiles of the ileum, colon, LM, and liver were evaluated as a shotgun approach to examine proteins and pathways that may be up- or down-regulated due to sCTC supplementation. Of the proteins identified across all tissues examined, 65 protein abundances were significantly different (*P* < 0.05) between sCTC and CON pigs (**Fig 1, Table 6**). In the ileum, 2 proteins were of lower abundance and 6 proteins were of greater abundance in sCTC pigs compared with CON pigs. These proteins were primarily involved with biological processes including metabolism and transport (**Fig 2**).

**Table 6:** Significantly different protein abundances in the ileum, colon, longissimus muscle, and liver of pigs in either control (CON) or sub-therapeutic chlortetracycline (sCTC; 40 ppm) treatments.

Description	UniProt ID	Log_2_ (CTC/CON)	*P*-value
**Ileum**			
Cofilin-2	Q5G6V9	-1.225	0.019
Ubiquitin-conjugating enzyme E2 D2	Q06AA9	-0.744	0.030
Arachidonate 15-lipoxygenase	P16469	1.721	0.012
Dolichyl-diphosphooligosaccharide-protein glycosyltransferase subunit 1	Q9GMB0	0.833	0.039
Dystrophin	Q5GN48	0.383	0.035
Ras-related protein Rab-14	Q52NJ6	0.567	0.012
Sodium/glucose cotransporter 1 (Fragment)	P26429	1.446	0.017
Sodium/potassium-transporting ATPase subunit alpha-2	D2WKD8	0.693	0.035
**Colon**			
60S ribosomal protein L15 (Fragment)	P79324	-0.793	0.017
60S ribosomal protein L6	Q2YGT9	-0.939	0.021
6-phosphogluconate dehydrogenase, decarboxylating (Fragment)	P14332	-0.977	0.024
ADP/ATP translocase 3	Q6QRN9	-1.149	0.018
Aspartate aminotransferase, mitochondrial	P00506	-0.771	0.012
Citrate synthase, mitochondrial	P00889	-0.568	0.043
Coatomer subunit beta	D2SW95	-0.846	0.048
Cytochrome C oxidase subunit 2	P50667	-1.249	0.023
Cytochrome C oxidase subunit 4 isoform 1, mitochondrial (Fragment)	Q95283	-0.875	0.048
Dolichyl-diphosphooligosaccharide-protein glycosyltransferase subunit 2	Q9GL01	-0.938	0.031
Epithelial cell adhesion molecule	Q75QW1	-0.725	0.027
Malate dehydrogenase, cytoplasmic	P11708	-0.607	0.045
Microsomal glutathione S-transferase 1	P79382	-1.181	0.036
Myosin-11 (Fragments)	P81271	-0.737	0.045
NADH-cytochrome b5 reductase 3 (Fragment)	P83686	-0.905	0.039
Prophenin-2	P51525	-1.676	0.037
Ras-related protein Rab-5A	Q06AU6	-0.815	0.021
Serum albumin	P08835	-0.641	0.021
Signal transducer and activator of transcription 1	Q764M5	-0.684	0.025
Tubulin alpha-1B chain	Q2XVP4	-0.607	0.036
Voltage-dependent anion-selective channel protein 2	Q9MZ15	-0.833	0.045
ATP synthase subunit f, mitochondrial	Q95339	-0.960	0.045
Ras-related protein Rab-14	Q52NJ6	-0.966	0.045
Phostensin	Q767M0	1.124	0.043
Short-chain specific acyl-CoA dehydrogenase, mitochondrial	P79273	0.564	0.041
**Longissimus muscle**			
Elongation factor 1-gamma (Fragment)	Q29387	-0.793	0.001
Malate dehydrogenase, cytoplasmic	P11708	-0.458	0.049
**Liver**			
40S ribosomal protein S12	P46405	0.251	0.025
40S ribosomal protein S20	A1XQU9	0.314	0.003
40S ribosomal protein S3	Q0Z8U2	0.302	0.033
40S ribosomal protein SA	Q4GWZ2	0.230	0.031
60S ribosomal protein L11	Q29205	0.243	0.039
60S ribosomal protein L29	Q95281	0.397	0.021
60S ribosomal protein L32	Q6QAT0	0.301	0.004
Apolipoprotein A-I	P18648	0.318	0.038
Bifunctional epoxide hydrolase 2	Q6Q2C2	0.437	0.046
Cystathionine gamma-lyase	Q19QT7	0.341	0.029
Cytochrome c oxidase subunit 5B, mitochondrial	Q5S3G4	0.389	0.023
Dihydropteridine reductase	Q8MJ30	0.308	0.022
Elongation factor 1-beta	P29412	0.303	0.039
Elongation factor 1-gamma (Fragment)	Q29387	0.324	0.005
Fructose-1,6-bisphosphatase 1	P00636	0.331	0.023
Gelsolin (Fragment)	P20305	0.853	0.002
Glucose-6-phosphate isomerase	P08059	0.312	0.018
Hemoglobin subunit alpha	P01965	0.555	0.046
Hemoglobin subunit beta	P02067	0.651	0.028
Hemopexin	P50828	0.609	0.016
High mobility group protein B1	P12682	0.502	0.017
High mobility group protein B2	P17741	0.557	0.048
Metallothionein-2A	P79379	1.170	0.012
Peroxiredoxin-2 (Fragment)	P52552	0.197	0.042
Prelamin-A/C	Q3ZD69	0.358	0.013
Serine/threonine-protein phosphatase 2A 65 kDa regulatory subunit A beta isoform (Fragment)	P54613	0.330	0.034
Serotransferrin	P09571	0.505	0.028
Serum albumin	P08835	0.455	0.011
Thymosin beta-4	Q95274	0.780	0.010
Transitional endoplasmic reticulum ATPase	P03974	0.192	0.021

In the colon, 23 proteins were of decreased abundance and 2 were of greater abundance in sCTC pigs compared with CON pigs (**Table 6**). Proteins of decreased abundance, while although having highly varied biological functions, were again primarily involved with metabolism and transport (**Fig 2**). Proteins of decreased abundance included ADP/ATP translocase 3, several subunits of cytochrome c oxidase, and malate dehydrogenase.

In the LM, 2 proteins were of decreased abundance in sCTC pigs compared with CON pigs (**Table 6**). These proteins, elongation factor 1-gamma and malate dehydrogenase, were both involved with metabolic processes (**Fig 2**).

In the liver, 30 proteins were of greater abundance in sCTC pigs compared with CON pigs (**Table 6**). These proteins were involved with metabolism, regulation of biological processes, response to stimulus, and transport (**Fig 2**). Many of these proteins were ribosomal proteins, and several proteins involved with energy generation were of increased abundance. The most differentially abundant liver protein was metallothionein-2A, a metal binding protein that had a log_2_ change of 1.17.

## Discussion

Sub-therapeutic levels of tetracyclines have been widely used in animal agriculture, largely due to their ability to consistently improve growth performance [2]. In the current study, ADG was improved 26% by sCTC (40 ppm), which is a larger increase than what is typically observed in a research setting [17–19]. Interestingly, feed efficiency did not differ in the current study, which differs from much of the historical literature demonstrating that antibiotics improve feed efficiency [19]. However, much of this historical research demonstrating feed efficiency differences is rather old or uses therapeutic doses of antibiotics. The lack of feed efficiency improvement is consistent with more recent observations by Shen et al. [18] using sub-therapeutic CTC. Thus, the lack of feed efficiency differences in the current experiment may be due to more modern pig genetics, more sanitary housing conditions, or the dose of CTC utilized. Further, sCTC pigs were 4 kg heavier than CON pigs at the end of the study, re-emphasizing the impact sub-therapeutic levels of antibiotics can have on nursery pig growth performance and producer economic returns. However, due to consumer and government concerns regarding increasing antimicrobial resistance, AGPs are being phased out of use by production animal agriculture [3, 20]. With the loss of such a crucial production tool, the swine industry has raced to develop alternatives that mimic the growth promoting benefits of AGPs without the risk of increasing antimicrobial resistance. However, despite their heavy usage by the industry for over 50 years, the mode of action by which AGPs improve growth performance is still unclear. Most research that has investigated the impact of CTC on pig growth, metabolism, and microbiota has utilized a therapeutic dose of 400 ppm or greater [21–23], which is 10 times greater than the sub-therapeutic dose used in the industry and likely has different effects on the pig and intestinal microbiota. Thus, this experiment aimed to investigate the mode of sub-therapeutic, rather than therapeutic, concentrations of CTC on pig growth and metabolism.

It has been hypothesized that the improvement in growth observed by feeding AGPs such as sCTC occurs via modulating the microbiome of the host [11], although studies examining microbial changes due to sub-therapeutic antibiotic supplementation have inconsistent results [24–28]. Furthermore, most of these studies failed to report growth performance parameters, making it difficult to evaluate whether changes in microbial communities translate into improved growth performance and/or feed efficiency. In the current experiment, we examined cecal SCFA concentrations as a marker of microbial metabolism, as they are the major products of microbial fermentation [29]. We observed no differences in cecum SCFA concentrations. These data are supported by Shen et al. [18], who observed no changes in SCFA concentrations in the cecum, as well as the colon and rectum of pigs when fed 80 ppm sCTC. This may suggest cecal microbial metabolism is not affected by sCTC and likely not a large contributor to sCTC’s mode of action in augmenting nursery pig growth. However, CTC at 100 ppm has been shown to decrease fecal *Bacteriodetes* and increase *Escherichia* and *Shigella* microbial communities in growing pigs [30]. Additionally, 50 ppm CTC has been shown to shift microbial communities in the ileum of pigs, but growth performance was not presented [26]. Although sub-therapeutic CTC has been shown to modulate bacterial populations, most of these studies have failed to report augmented growth performance. Thus, the role these microbial shifts may play in modulating host intestinal function and integrity, as well as whole-body metabolism and growth, remains unclear.

An alternative, host-centric hypothesis regarding how sub-therapeutic antibiotics facilitate growth has also been proposed [31]. Although this hypothesis has received less attention, it is thought that AGPs act on the host via immunomodulation, decreasing the costs of supporting immune functions and allowing more nutrients to be allocated towards lean tissue accretion and growth [13, 31]. It is also hypothesized that growth promoting antibiotics may result in thinner villi lamina propria, which may allow for enhanced nutrient uptake into the bloodstream [2, 11, 32]. In order to examine if changes in the intestinal barrier are one of the mechanisms by which AGPs improve growth, we utilized modified Ussing Chambers to examine ex vivo intestinal barrier permeability and active nutrient transport in the ileum and colon. Increases in barrier permeability are associated with periods of inflammation, stress, and a reduction in the capacity for growth [33]. On the contrary, increases in active transport or transporter abundance are associated with the uptake of more nutrients from the intestinal lumen, increasing capacity for growth. In the current experiment, sCTC treatment did not appear to have an impact on small or large intestinal integrity, as assessed by ileum and colon TERs. Similarly, the active transport of glucose and glutamine across the ileum was not different between sCTC and CON pigs. Furthermore, no differences in tight junction protein and nutrient transporter gene abundance were reported. Thus, it appears unlikely that changes to intestinal barrier permeability and active nutrient transport function explained the increased growth associated with sCTC treatments. However, it is noted these measurements were only collected at the end the study, and any changes in these parameters may have stabilized by the end of the 35 day trial.

In line with the immunomodulatory hypothesis, the abundance of the antimicrobial peptide BD2 tended to be reduced in sCTC pigs compared with CON pigs. Beta-defensin 2 has been shown to have antimicrobial activity in the intestine [34] and likely possesses pro-inflammatory and chemoattractant effects [35], thus a reduction in BD2 may suggest a lower level of inflammation at the ileal epithelium. Similarly, the chemoattractant CCL2 had reduced abundance in sCTC pigs, suggesting that AGPs may act to reduce inflammation at the ileum. These localized reductions in inflammation may translate into a global reduction in inflammatory markers, which has been observed in the serum of pigs treated with sub-therapeutic oxytetracycline [36]. Reductions in circulating inflammatory markers such as cytokines, which are known to reduce appetite [37], may help explain the increased feed intake and thus increased growth of sCTC pigs.

Several more important findings from this study are that the proteomes of the ileum, colon, skeletal muscle, and liver were all found to be altered by sCTC addition to the diet. Further, protein changes were tissue specific, but consistently involved with metabolism. Interestingly, differentially abundant proteins showed a clear trend towards downregulation in the colon, and a clear trend towards upregulation in the liver. Only 2 proteins were differentially abundant in the LM, suggesting that LM metabolism is largely unaffected by sCTC. Downregulated proteins in the colon were largely related to metabolism and transport, such as ADP/ATP translocase, malate dehydrogenase, and electron transport chain components cytochrome c oxidase subunit 2 and subunit 4. This suggests that the colon is less metabolically active in pigs fed sCTC, perhaps reflecting a large intestinal energy sparing mechanism. Metabolism related proteins in the liver were of greater abundance, such as the glycolytic protein glucose-6-phosphate isomerase, the gluconeogenic enzyme fructose-1,6-bisphosphatase, and cytochrome c oxidase. Additionally, elongation factors 1-beta and 1-gamma, proteins that facilitate translation elongation, were also of greater abundance in the liver. The liver is a highly metabolic organ in the pig [38], thus an increase in metabolism related proteins is unsurprising, and likely related to the overall faster growth rate of the sCTC pigs. An upregulation in genes related to lipogenesis and triglyceride synthesis have been observed in the liver of mice dosed with sub-therapeutic levels of various antibiotics, including penicillin and chlortetracycline [39], demonstrating that sub-therapeutic antibiotics directly modulate the host and furthers the hypothesis that the growth promoting characteristics of AGPs are partially a result of the host response. However, these changes in metabolism and growth may also just be a function of higher feed intakes, which we and others [17, 18] have observed to be increased as a result of sCTC supplementation.

In conclusion, in-feed sub-therapeutic CTC supplementation at 40 ppm increased nursery pig growth performance, primarily due to an increase in feed intake. Ileum integrity and function, and cecal microbial metabolism do not appear to explain the differences observed. However, changes in several ileum transcripts suggest that inflammation may be reduced in sCTC pigs. Further, the changes seen in the proteomic profile suggest that the sub-therapeutic mode of action of AGPs may include post-absorptive changes and warrants further investigation.

## Supporting information

S1 Table Primer sequences. (DOCX)

S1 Dataset Raw data files. (ZIP)
