# Supplementary material for: Evaluating nursery pig responses to in-feed sub-therapeutic antibiotics
Source: PLoS One. 2019 Apr 26;14(4):e0216070. doi: 10.1371/journal.pone.0216070 (PMC6485771; doi:10.1371/journal.pone.0216070)
Supplement: S1 Table — (DOCX) [file pone.0216070.s001.docx]

| **Gene** | **Sense (5'-3') - forward** | **Antisense (5'-3') - reverse** |
| --- | --- | --- |
| ACTB | CCAACATTGGTTATGGGAGCAA | GGAAGAGACGTTGTGAGCAA |
| BD2 | GCTGACTGTCTGCCTCCTCT | CAGGTCCCTTCAATCCTGTT |
| CASP3 | ACTGGCAAACCCAAACTTTTCA | GTCCCACTGTCCGTCTCAA |
| CASP6 | CGCAGACTTCCTCATGTGTTAC | ATGTACCAGGAGCCGTTCAC |
| CCL2 | AAGAGTCACCAGCAGCAAGT | GGGTTCTGCACAGATCTCCTT |
| CLDN2 | AGGCCTCCTGGGCTTCAT | GGAGTAGAAGTCCCGCAGGAT |
| CLDN3 | TTGCATCCGAGACCAGTCC | AGCTGGGGAGGGTGACA |
| CLDN4 | GTATCATCCTGGCCGTGCTA | TTGGCGCTCTCATCATCCA |
| DEFB1 | ACCGCCTCCTCCTTGTATTC | GGTGCCGATCTGTTTCATCT |
| FABP1 | TGAACTCAACGGTGACATA | ATTCTCTTGCTGATTCTCTTG |
| IAP | AACCGCAGGACATTCCTTCA | TTCATGTCTGCCGGCTCAA |
| IL10 | GGAGAAGCTGAAGACCCTCA | CGGCCTTGCTCTTGTTTTCA |
| IL18 | CCATCTCTGTGCAGTGTAAGAA | GTCCAGGAACACTTCTCTGAAA |
| IL8 | GCACTTACTCTTGCCAGAACTG | CAAACTGGCTGTTGCCTTCTT |
| MUC2 | CTGTGTGGGGCCTGACAA | AGTGCTTGCAGTCGAACTCA |
| NFKB1 | TGTGAAGACCACCTCTCAGG | CTGTCACAGATGCTGTCATCC |
| OCLN | TCGTCCAACGGGAAAGTGAA | ATCAGTGGAAGTTCCTGAACCA |
| RELA | AATCAGCGCATCCAGACCAA | ACAGCATTCAGGTCGTAGTCC |
| RPL32 | CCAACATTGGTTATGGGAGCAA | GGAAGAGACGTTGTGAGCAA |
| SLC5A1 | GGCTGTTCCAACATTGCCTA | CAACATGACCGACAGCATCA |
| SLC5A8 | CGCAGATTCCTACTAACC | GATTGTCAGTTCCACCAT |
| SLCA2 | TCATCAGCTGGCCATTGTCA | GCTCATGATTGCCCAGGAGAA |
| TFF2 | GGGTCCCCTGGTGTTTCAA | ACCTCCATGACGCACTCC |
| TGFB1 | CATCGACATGGAGCTGGTGAA | GAGCCGAAGCTTGGACAGAA |
| TLR2 | CGGCTTCCAAGGATGGAGAA | AGACCCATGCTGTCCACAAA |
| TLR3 | GTCCAACTCAATCCAGAAGATTCAAA | AGTTGGAGCTGCGTTCCTAA |
| TLR4 | TGGTGTCCCAGCACTTCATA | CGGCATGACTCCTCAGAAAC |
| TNFA | CTGGCCCCTTGAGCATCA | GGGCTTATCTGAGGTTTGAGAC |
| YWHAZ | AGACAGCACGCTAATAATGCA | CCTGCTTCAGCTTCATCTCC |

**Supplementary table 1**. Primer sequences
